# Evolutionary Conservation in Protein–Protein Interactions and Structures of the Elongator Sub-Complex ELP456 from *Arabidopsis* and Yeast

**DOI:** 10.3390/ijms25084370

**Published:** 2024-04-15

**Authors:** Sang Eun Jun, Kiu-Hyung Cho, Raffael Schaffrath, Gyung-Tae Kim

**Affiliations:** 1 Department of Molecular Genetics, Dong-A University, Busan 49315, Republic of Koreakhcho68@gmail.com (K.-H.C.); 2 Gyeongbuk Institute for Bioindustry, Andong 36618, Republic of Korea; 3 Institut für Biologie, Fachgebiet Mikrobiologie, Universität Kassel, Heinrich-Plett-Str. 40, 34132 Kassel, Germany; schaffrath@uni-kassel.de; 4 Graduate School of Applied Bioscience, Dong-A University, Busan 49315, Republic of Korea

**Keywords:** *Arabidopsis*, Elongator, ELP456 sub-complex, protein homology

## Abstract

The Elongator complex plays a pivotal role in the wobble uridine modification of the tRNA anticodon. Comprising two sets of six distinct subunits, namely, Elongator proteins (ELP1-ELP6) and associated proteins, the holo-Elongator complex demonstrates remarkable functional and structural conservation across eukaryotes. However, the precise details of the evolutionary conservation of the holo-Elongator complex and its individual sub-complexes (i.e., ELP123; ELP456) in plants remain limited. In this study, we conducted an in vivo analysis of protein–protein interactions among *Arabidopsis* ELP4, ELP5, and ELP6 proteins. Additionally, we predicted their structural configurations and performed a comparative analysis with the structure of the yeast Elp456 sub-complex. Protein–protein interaction analysis revealed that AtELP4 interacts with AtELP6 but not directly with AtELP5. Furthermore, we found that the *Arabidopsis* Elongator-associated protein, Deformed Roots and Leaves 1 (DRL1), did not directly bind to AtELP proteins. The structural comparison of the ELP456 sub-complex between *Arabidopsis* and yeast demonstrated high similarity, encompassing the RecA-ATPase fold and the positions of hydrogen bonds, despite their relatively low sequence homology. Our findings suggest that *Arabidopsis* ELP4, ELP5, and ELP6 proteins form a heterotrimer, with ELP6 serving as a bridge, indicating high structural conservation between the ELP456 sub-complexes from *Arabidopsis* and yeast.

## 1. Introduction

The Elongator complex was initially identified from yeast (*Saccharomyces cerevisiae*) in association with elongating RNA polymerase II [1]. Among its six subunits (Elp1–Elp6), yeast Elp3 (ScElp3) is catalytically active as an acetyltransferase, contributing to protein and, importantly, to tRNA modification [2,3,4,5]. Beyond transcription and potential histone acetylation, the Elongator complex is thus implicated in diverse cellular processes, including exocytosis regulation and roles in telomeric gene silencing and DNA repair [6,7,8]. Notably, its involvement in uridine modifications at the tRNA anticodon wobble position (U34) has been shown to impact growth, development, and stress responses in yeast [9,10,11,12].

In the context of human diseases, Elongator defects in tRNA modification have been linked to neurodegenerative/neurodevelopmental syndromes [13,14,15,16,17], emphasizing the significance of Elongator’s role in uridine modification at the tRNA wobble position [2,11,18]. Despite its association with various cellular processes, the role that Elongator plays in mRNA translation regulation, particularly through tRNA modification in eukaryotes, remains poorly understood.

Structurally, the Elongator complex consists of two sub-complexes: a catalytic sub-complex (ELP123) and an accessory sub-complex (ELP456), along with an Elongator-associated protein DRL1/Kti12 that provides tRNA binding and regulation of Elongator activity in yeast [1,19,20,21,22]. The hexameric ring structure of the yeast Elp4 (ScElp4), Elp5 (ScElp5), and Elp6 (ScElp6) proteins, forming a RecA-ATPase-like fold, is pivotal for the assembly of the Elp456 sub-complex [22,23,24,25]. The holo-Elongator complex is believed to involve two copies of the Elp123 sub-complex interacting asymmetrically with the hexameric ring of the Elp456 sub-complex [24,25,26,27]. This conformation is crucial for tRNA association and dissociation at the Elp456 ring and the transfer of tRNA molecules between sub-complexes [26].

In *Arabidopsis*, Elongator loss-of-function mutants exhibit distinctive phenotypic traits and affect the expression of genes involved in various processes, including auxin biosynthesis, chromatin assembly, and responses to abiotic stresses [28,29,30]. Functional distinctions between the two ELP sub-complexes have been observed, with mutants of *elp1* and *elp2* showing drought tolerance compared to *elp4* and *elp6* mutants [28,29]. Furthermore, studies on tRNA wobble position modifications in *Arabidopsis* mutants emphasize a critical role of Elongator in plant development and stress responses [31,32]. Notably, loss-of-function mutations in *ELP4* and an *Arabidopsis* Elongator-associated protein, *DRL1*, result in developmental abnormalities, including impaired shoot apical meristem activity, disrupted cell proliferation, inhibited root elongation, and altered leaf morphology, characterized by trumpet-like or narrow leaves, epinastic curling, and abaxialization of the adaxial side [31,33,34].

Involvement of the Elongator complex in tRNA modification underscores its pivotal role in mRNA translation elongation. However, the mechanisms of potential translational regulation mediated by Elongator-associated tRNA wobble U34 modification remain largely unknown in plants. Considering the influence of tRNA modification on the structure of anticodon nucleosides and its reliance on conformational changes, comprehending the structural–functional relationships of the Elongator complex is crucial. We hypothesized that *Arabidopsis* AtELP4 and AtDRL1 share evolutionarily conserved functions with species-specific variations compared to their yeast homologs. This hypothesis is supported by evidence of partial complementation observed when *Arabidopsis* ELP4 or DRL1 are expressed in yeast *elp4* or *kti12* mutants [31,34,35]. Therefore, gaining structural insights into Elongator proteins and sub-complexes has the potential to shed light on both the evolutionary conservation and diversification of functions within the Elongator complex [31,34,35]. Our study aims to provide structural insights into Elongator proteins and sub-complexes, unveiling the conserved structure of the *Arabidopsis* ELP456 sub-complex through yeast two-hybrid assays and protein structure prediction analysis. Comparative analysis with the yeast and *Arabidopsis* Elp456 sub-complex reveals a remarkably similar structure, suggesting evolutionary conservation in the functional attributes of the Elongator complex from a simple to a multicellular model in eukaryotes.

## 2. Results

### 2.1. AtELP4 Interacts with AtELP6 but Not Directly with AtELP5

**Figure 1:**
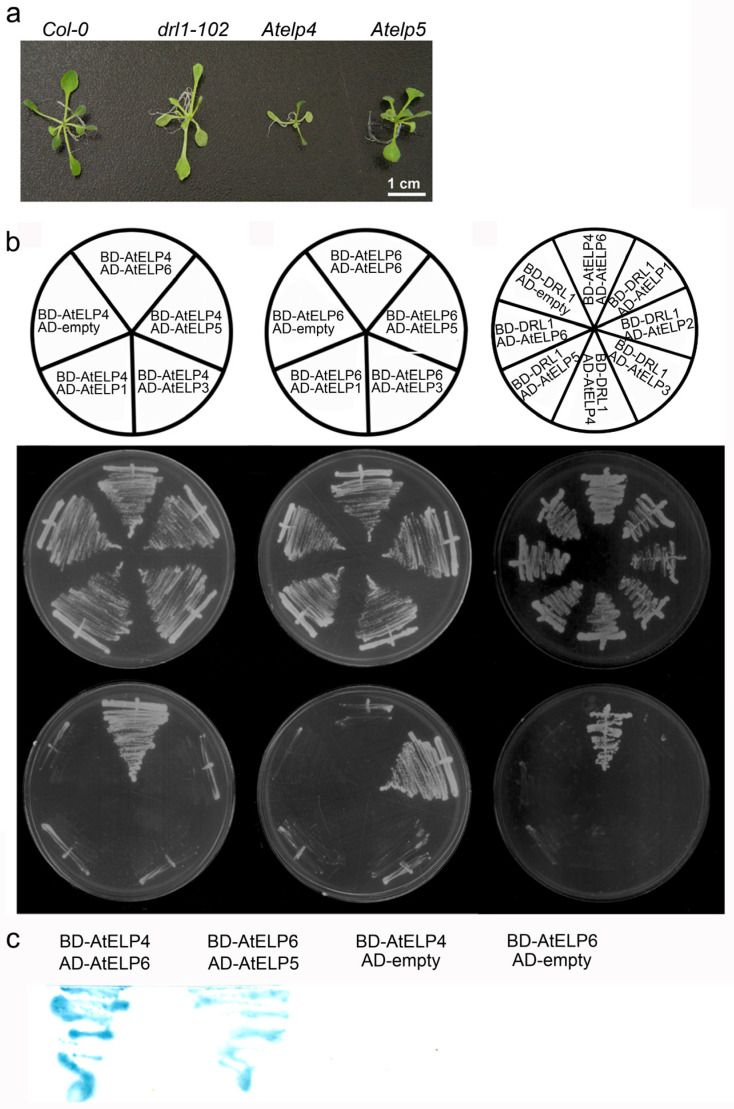
Phenotypes of *Arabidopsis* Elongator protein loss-of-function mutants and protein–protein interactions between Elongator subunits. (**a**) Plant phenotypes of wild-type and *drl1-102*, *Atelp4*, and *Atelp5* single mutants at 21 days after sowing (DAS). (**b**) Yeast two-hybrid interaction assays between Elongator proteins. (**c**) X-gal assay after growth on selective media. The annotation indicates bait (DNA-binding domain, BD in pDEST32 destination vector) and prey (activation domain, AD in pDEST22 destination vector) constructs used in panels (**b**,**c**). Co-transformation of pDEST22 empty vector with pDEST32 containing AtELP4 or AtELP6 was used as a negative control. Growth on SD media deficient in leucine and tryptophan (SD/-Leu/-Trp) was used to confirm the co-transformation of prey and bait (middle row). Growth on selective media, SD media lacking leucine, tryptophan, and histidine (SD/-Leu/-Trp/-His) and containing 10 mM 3-amino-1,2,4-triazole (3-AT), to test interactions among AtELPs (lower row).

In this study, we examined the impact of the loss of function of *Arabidopsis* ELP5 (*Atelp5*) on plant morphology, which revealed slight alterations compared to the wild type (Figure 1a). Conversely, mutants of the *Arabidopsis DRL1* gene (*drl1-102*) and *ELP4* gene (*Atelp4*) displayed distinct phenotypes, characterized by narrow leaves with a downward curling phenotype [31]. Our previous investigations into *drl1-102* and *Atelp4* single mutants, as well as double mutants, demonstrated a reduction in leaf width, by cell proliferation and expansion, and an increased proportion of the intercellular airspace between palisade mesophyll cells [31,36], suggesting that *AtELP4* and *AtDRL1* are involved in the same genetic pathway.

Our previous research has indicated partial functional conservation between AtDRL1 and AtELP4 and their respective yeast homologs, ScKti12 and ScElp4, as evidenced by traits such as temperature and caffeine sensitivity [31,34]. However, the amino acid sequences of AtELP4, AtELP5, and AtELP6 share relatively low identity with their yeast counterparts, with 22.65%, 23.13%, and 20.99%, respectively (Table 1). Thus, it is important to investigate the structural aspects of the *Arabidopsis* Elongator complex followed by comparison with the yeast counterpart complex.

To confirm interactions between individual Elongator subunits in *Arabidopsis*, we performed a yeast two-hybrid analysis. In this assay, *Arabidopsis* ELP4 and ELP6 were fused to the Gal4 DNA-binding domain, serving as bait, while AtELP1, AtELP3, AtELP5, or AtELP6 were fused to the Gal4 activation domain, acting as prey. Co-transformation on selective media revealed that AtELP6 interacted with both AtELP4 and AtELP5, whereas AtELP4 did not interact with AtELP5 (Figure 1b). In particular, AtELP4 exhibited a strong interaction with AtELP6 but not with AtELP1, AtELP3, or AtELP5 (Figure 1b left column). On the other hand, AtELP6 did not display interactions with AtELP1, AtELP3, or itself (Figure 1b, middle column). The β-galactosidase assay further supported the strong interaction between AtELP4 and AtELP6 and between AtELP5 and AtELP6 (Figure 1c). Our results of the protein–protein interactions among *Arabidopsis* Elongator subunits in this study suggest that AtELP4, AtELP5, and AtELP6 may form a heterotrimer, with *AtELP6* potentially acting as a bridging component that connects the other subunits.

### 2.2. AtDRL1 Does Not Directly Interact with Each Subunit of the AtELP456 Sub-Complex

Previous genetic studies have revealed that *DRL1*, an ortholog of the yeast Elongator-associated protein Kti12, and *AtELP4* genes are involved in same genetic pathway of leaf development. To examine the physiological correlation between ELP proteins and DRL1, we investigated the protein–protein interactions between them. Our analysis showed that DRL1 exhibited a weak interaction with AtELP5 but did not interact with the other Elongator proteins (Figure 1b, right column). This result suggests that DRL1 may not interact with individual AtELP proteins but rather forms an interaction with the *Arabidopsis* Elongator holo-complex. Notably, our observations were also supported by previous research by demonstrating that the *S. cerevisiae* DRL1 homolog Kti12 (ScKti12) was associated with Elongator in a salt-labile manner [37]. Their study showed that ScKti12 was not detected in highly purified yeast holo-Elongator complexes. In addition, we previously showed that ScKti12 was a PSTK-like ATPase and tRNA-binding protein required for Elongator to function in tRNA modification and interacted with the yeast Elongator subunit Elp1 [20,35].

### 2.3. Structure Analysis of Arabidopsis ELP4, ELP5, and ELP6 Subunits

**Figure 2:**
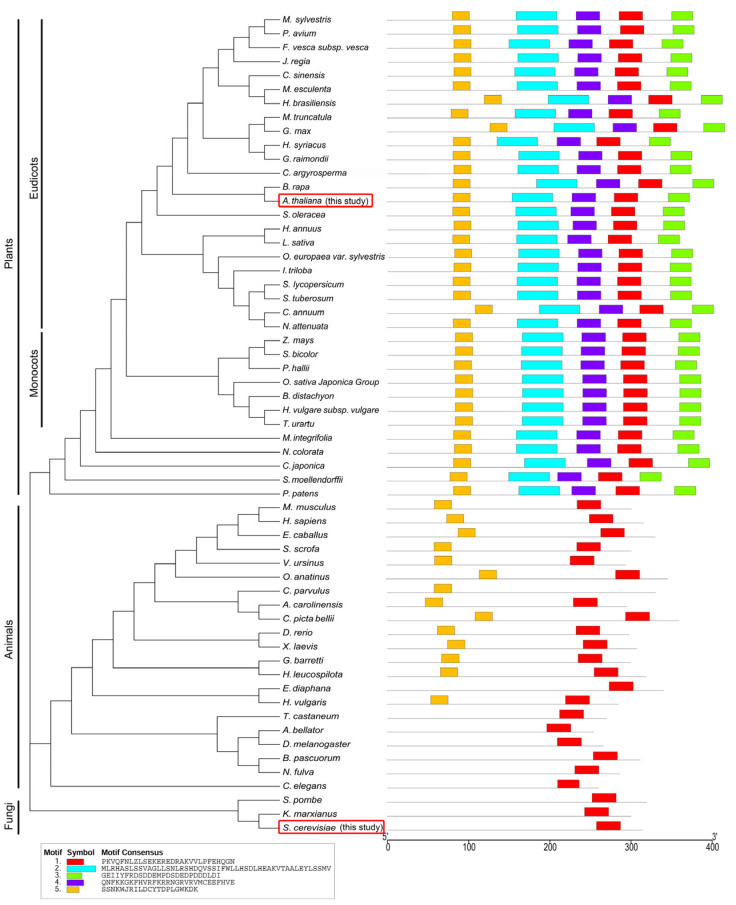
The phylogeny and conserved motif analysis. ELP5 proteins were classified into three groups, plants, yeast, and animals, and their motifs are represented by distinct colored boxes. Sequence of conserved motifs is indicated in the open box. ELP5 from organisms used in this study is highlighted within a red open box.

Despite the low amino acid sequence identity observed between ELP proteins in *Arabidopsis* and yeast, as indicated above (Table 1), our prior research demonstrated that ELP4 exhibits a high degree of functional conservation and a diverse distribution of motifs across eukaryotes [31]. Additionally, phylogenetic analysis and an examination of conserved motifs in eukaryotic ELP5 revealed its subclassification into three groups: yeast, animals, and plants. Furthermore, ELP5 not only falls within distinct kingdoms but also displays kingdom-specific motif distribution (Figure 2). Motif 1 (Figure 2, red box) was commonly involved in ELP5 proteins from all of plants, yeast, and animals, while motif 5 (yellow box) was present in ELP5 proteins of plants and animals. Motif 2 (Figure 2, cyan box), motif 3 (Figure 2, green box), and motif 4 (Figure 2, violet box) appeared only in ELP5 from plants. ScELP5 also possesses the common structural elements in RecA-ATPase, like ScELP4 and ScELP6 [25]. These findings suggest that ELP5 has undergone unique evolutionary processes while still maintaining basal representative functions.

Our previous studies have also reported that the AtELP456 sub-complex plays a pivotal role in leaf dorsoventrality [31,36], potentially through tRNA modification processes. To ascertain the structural roles of the AtELP456 sub-complex within the assembly of the holo-complex, we conducted in silico analyses, comparing sequence-based predicted structures of *Arabidopsis* ELPs with experimentally determined yeast ELP structures, PDB ID 4A8J [38].

**Figure 3:**
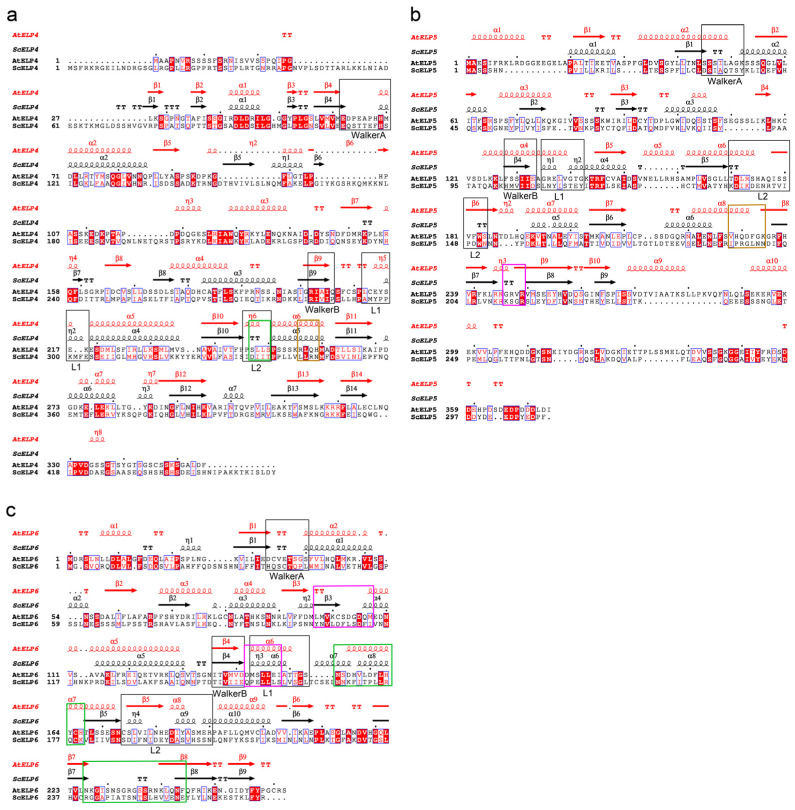
Structure-based sequence alignment of ELP subunits between *Arabidopsis* and yeast. To compare the structure of ELP456 proteins between *Arabidopsis* and yeast, crystal structures of the yeast Elp456 sub-complex (PDB ID 4A8J; [38]) were utilized. (**a**) AtELP4. (**b**) AtELP5. (**c**) AtELP6. Primary and secondary structure alignment: Primary structure alignment was performed using ClustalW [40] in MEGA7 software (ver. 7) [41], and aligned primary and corresponding secondary structure elements of each ELP subunit were generated using ESPript3.0 software (https://espript.ibcp.fr/ESPript/ESPript/, accessed on 21 September 2023); [42]). Every tenth residue of *Arabidopsis* ELP is indicated with a dot. Strictly identical residues in aligned sequences are shown as white characters in red boxes, and while highly similar amino acid residues (>80%) are depicted as black bold characters in yellow boxes. Secondary structures of ELPs from *Arabidopsis* (red color) and yeast (black color) are presented on top. α-Helices, β-sheets, and 3_10_(η)-helices are marked with α, β, and η, respectively, and numbered accordingly. β-Turns and α-turns are represented by TT and TTT, respectively. The black boxes indicate regions conserved with Walker A, Walker B, L1, and L2 motifs in *E. coli* RecA. The colored boxes indicate shared regions of hydrogen bonds with ELP4 (magenta), with ELP5 (green), and with ELP6 (brown).

**Figure 4:**
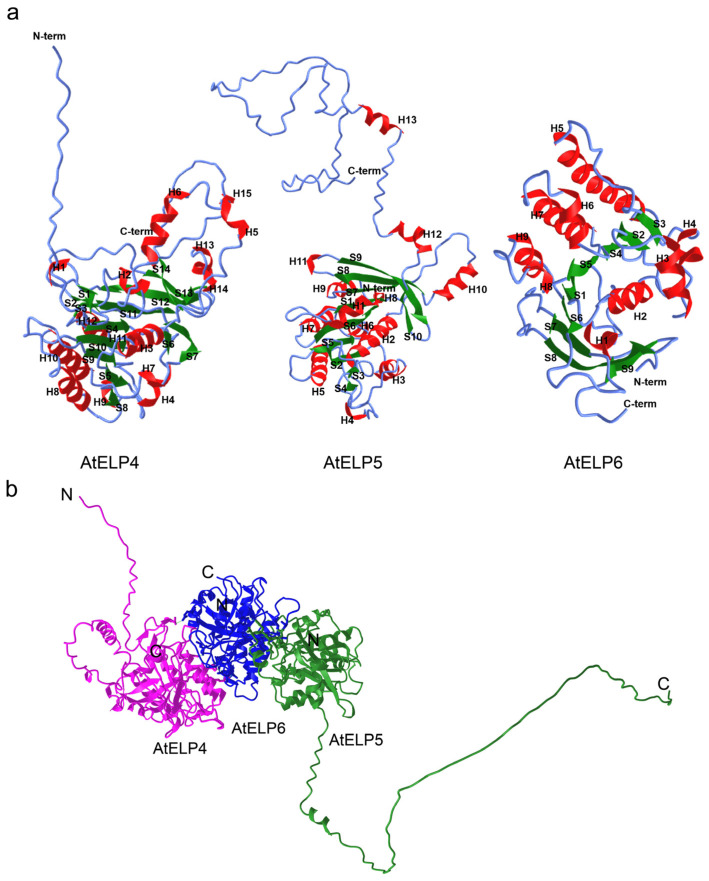
Predicted tertiary structure of the *Arabidopsis* ELP456 sub-complex. (**a**) Individual subunit structures: AtELP4 (**left**), AtELP5 (**middle**), and AtELP6 (**right**). α-Helices are indicated in red (H), β-sheets in green (S), and coils as blue lines. (**b**) Structure of the AtELP456 sub-complex. Magenta represents AtELP4, green represents AtELP5, and blue represents AtELP6. The letters N and C indicate the N- and C-terminal ends of each protein.

The predicted tertiary structure of AtELP4 revealed a total of 15 helical structures, including 7 α-helices (α1–7), 8 3_10_(η)-helices (η1–8), and 14 β-sheets (β1–14) (Figure 3a and Figure 4a, left). In the case of AtELP5, its predicted tertiary structure comprised 13 helical structures, encompassing 10 α-helices (α1–10), 3 3_10_(η)-helices (η1–3), and 10 β-sheets (β1–10) (Figure 3b and Figure 4a, middle). The predicted tertiary structure of AtELP6 featured nine α-helices (α1–9) and nine β-sheets (β1–9) (Figure 3c and Figure 4a, right). Consequently, it is evident that *Arabidopsis* ELP4, ELP5, and ELP6 share similar tertiary structures characterized by helices surrounding centrally located parallel and spiral β-sheets.

Our yeast two-hybrid experiment revealed direct interactions between ELP4 and ELP6 and between ELP5 and ELP6, indicating the formation and presence of the AtELP456 sub-complex (Figure 1). To further elucidate the structural aspects of this complex, we performed structure predictions using AlphaFold Multimer [39]. The predicted structure of the AtELP456 sub-complex exhibited high confidence, with a pLDDT score of 75.8, a pTMscore of 0.792, and an ipTM of 0.768. This structure revealed a tripartite complex configuration with AtELP6 positioned at the center. Additionally, AtELP4 and AtELP5 were situated on both sides, resembling wings (Figure 4b), thus providing support for the observed AtELP4–AtELP6 and AtELP5–AtELP6 interactions in vivo (Figure 4b). The calculated distances between AtELP4 and AtELP6, between AtELP5 and AtELP6, and between AtELP4 and AtELP5 were 3.31 nm, 4.79 nm, and 6.97 nm, respectively (Figure 4b).

**Figure 5:**
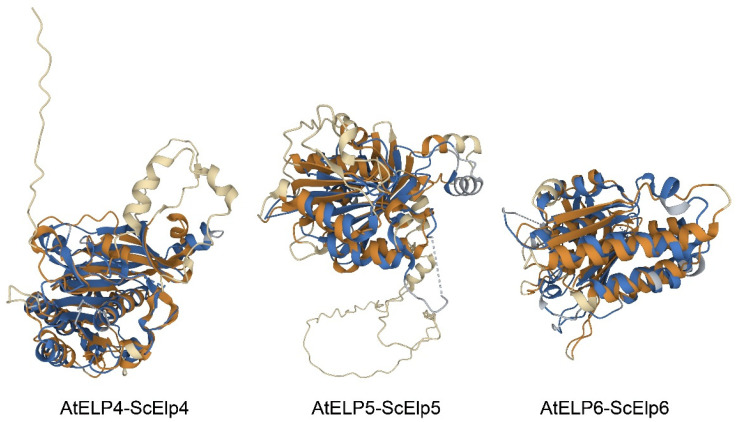
Superposition of tertiary structures of ELP4, ELP5, and ELP6 proteins between *Arabidopsis* and yeast. Protein structures are depicted as ribbon plots. Superpositions were created using the Pairwise Structure Alignment tool available on the RCSB PDB server. *Arabidopsis* ELP proteins are depicted in brown, while yeast Elp subunits are shown in blue. Aligned residues are displayed in brightly colored regions, while unaligned portions are shown in the lighter shades of the same color.

On the other hand, Glatt et al. [24] and Lin et al. [25] proposed the potential presence of P-loop ATPase regions in the ScElp4, ScElp5, and ScElp6 subunits, which bridge the β2 strand and α1 strand for ATP binding. Based on this proposal, we analyzed primary and secondary structure alignments to identify key sequences of ATPase, including WalkerA (P-loop), WalkerB, L1, and L2 motifs in *Arabidopsis* ELP4, Elp5, and ELP6 subunits (Figure 3). Potential P-loop regions were found in AtELP4 and AtELP6, which are situated between β-sheet and helix elements (between the β4 strand and α2 strand in AtELP4, between the β3 strand and η1 strand in AtELP5, and between the β1 strand and α2 strand in AtELP6) (Figure 3a,c). However, these regions lacked consensus residues associated with the P-loop/WalkerA motif (GXXXXG.KT motif), which is an essential motif in RecA ATPase. This structural alignment suggests that the AtELP456 sub-complex adopts a P-loop ATPase-like fold, even though it lacks the ATPase key sequence, similar to the yeast Elp456 sub-complex (PDB ID 4A8J) [38]. To assess the structural similarity, we compared the AlphaFold2-predicted structure of *Arabidopsis* ELP subunits with the experimental crystal structure of yeast Elp subunits (PDB ID 4A8J; [38]) using jFATCAT (rigid) in the Pairwise Structure Alignment tool on the RCSB PDB server (https://www.rcsb.org/alignment, Piscataway, NJ, USA, accessed on 24 October 2022). The pairwise structure alignment between AtELP4 and ScElp4 (PDB ID 4A8J chain A) revealed 261 equivalent residue pairs, a Template Modeling score (TM-score) of 0.65 and a root mean square deviation (RMSD) of 3.08 (Figure 5, left, and Table 2). For AtELP5 and ScElp5 (PDB ID 4A8J chain B) structure alignment, we observed 187 equivalent residue pairs, a TM-score of 0.43, and an RMSD of 3.05 (Figure 5, middle, and Table 2). Finally, the structure alignment between AtELP6 and ScElp6 (PDB ID 4A8J chain C) indicated 230 equivalent residue pairs, a TM-score of 0.74, and an RMSD of 3.05 (Figure 5, right, and Table 2). Typically, a TM-score higher than 0.5 suggests a similar overall fold [43]. Our findings, along with the high reference/target coverage indicative of a significant fraction of matched residues in the superposition (Figure 5), support the idea that the ELP456 sub-complex, particularly ELP4 and ELP6, maintains a highly conserved structure between *Arabidopsis* and yeast despite the low sequence homology.

### 2.4. Interaction Prediction between AtELP4, AtELP5, and AtELP6 Proteins

Because hydrogen bonds play a crucial role in directing interactions for protein folding and ligand recognition [44], we identified potential hydrogen bonds with a default distance cutoff of less than 0.38 nm between AtELP4 and AtELP6 and between AtELP5 and AtELP6 based on the AtELP456 structure predicted by AlphaFold Multimer (Table 3 and Table 4). In the AtELP4–AtELP6 interface, seven hydrogen bonds were predicted, involving Arg256, His259, Asn32, Gln180, and Ser231 of AtELP4 and Glu147, His181, Asp183, Glu250, Lys100, and Leu97 of AtELP6 (Figure 3a,c and Table 3). Remarkably, five out of these seven hydrogen bonds were found within residues situated between helices α5 and α6 of AtELP4. In the AtELP5-AtELP6 interface, we identified eleven hydrogen bonds, involving Trp100, Tyr94, Asp231, Phe232, Gln230, Val228, and His229 of AtELP5 and Arg119, Tyr164, His166, Ser170, Ser237, Asn239, Leu241, Asn243, and Tyr257 of AtELP6 (Figure 3b,c and Table 3). Notably, seven out of these eleven hydrogen bonds were located within residues between helix α8 and β-sheet β8 of AtELP5.

To identify structural key residues or motifs in the ELP456 sub-complex, we compared the positions of hydrogen bonds among the ELP4, ELP5, and ELP6 proteins in *Arabidopsis* and yeast. The hydrogen bonds in the ELP4, ELP5, and ELP6 proteins appeared at identical positions when aligning the sequences of each *Arabidopsis* and yeast ELP protein (Figure 3). In ELP4, hydrogen bonds with ELP6 were formed identically on the α6 strand in *Arabidopsis* and the α5 strand in yeast. Hydrogen bonds in ELP6 with ELP4 were formed between the β3 strand and α5 strand in Arabidopsis and between the β3 strand and α4 strand in yeast (Figure 3a,c). Hydrogen bonds with ELP6 in ELP5 were observed between the α8 strand and β8 strand in *Arabidopsis* and between the α6 strand and β7 strand in yeast. Meanwhile, hydrogen bonds with ELP5 in ELP6 were observed in the α7 strand and between β7 and β8 in *Arabidopsis* and in the α8 strand and between the β7 strand and β8 strand in yeast (Figure 3b,c). Considering both position and structure, our findings suggest the potential significance of these regions to play key roles in assembling the ELP456 sub-complex.

It is important to note that yeast the Elp456 sub-complex was reported to form a homodimer of Elp456 heterotrimers (hexamer) with a ring-like structure [24,25]. While we could not model the hexamer structure, we predicted the interfaces between AtELP4 and AtELP5 that contribute to hexamer formation using AlphaFold Multimer. We found eleven hydrogen bonds in the AtELP4 and AtELP5 interface, involving Ser247, Ile270, Asp272, and Tyr284 of AtELP4 and Asn196, Val248, and Arg247 of AtELP5 (Figure 3a,b and Table 4). Hydrogen bonds between ELP5 and ELP4 were formed between the β10 strand and α6 in *Arabidopsis* and between the β10 strand and α5 in yeast. Meanwhile, hydrogen bonds with ELP4 in ELP5 were established between the β8 strand and β9 in *Arabidopsis* and between the β7 strand and β8 strand in yeast (Figure 3a,b). A comparison of the positions of these hydrogen bonds suggests that protein–protein binding sites between ELP456 proteins are conserved between *Arabidopsis* and yeast. Collectively, our computational and in vivo analyses suggest that the *Arabidopsis* ELP456 sub-complex forms a trimer through interactions involving AtELP4 and AtELP6, AtELP5 and AtELP6, and AtELP4 and AtELP5.

## 3. Discussion

The function of Elongator is evolutionarily conserved from archaea to eukaryotes, and the Elongator complex, consisting of six subunits (ELP1–ELP6), is structurally preserved across yeast, animals, and plants [45,46]. However, there is a notable disparity in the similarity of amino acid sequences between the subunits of yeast and plants, as illustrated in Table 1. Moreover, plant-specific domains have been identified in AtELP4 [31], AtELP5 (Figure 2), and Elongator interactor AtDRL1 [34], suggesting the acquisition of distinct functions during the course of evolution [34,35]. In previous work, we proposed that AtELP4 shares a conserved P-loop ATPase motif found in RecA and the synergistic role of AtELP4 and AtDRL1 in cell proliferation and leaf dorsoventrality [31]. Despite their genetic interaction, we observed that these two Elongator subunits do not directly interact in a yeast two-hybrid analysis (Figure 1). We previously demonstrated that ScKti12 acts as a PSTK-like ATPase and tRNA-binding protein essential for Elongator to function in tRNA modification. Furthermore, it could not be replaced by plant DRL1, despite DRL1 being expressed in ScKti12 mutant and interacting with yeast Elp1 [20,35]. Thus, we speculate that the incompatibility between yeast Kti12 and plant DRL1 likely stems from reasons other than mere protein–protein interaction. Taken together, despite the conserved function, this interaction apparently is species-specific and seems to have co-evolved species-specifically, as gene shuffle experiments showed incompatibility between yeast and plant Elongator populations.

In this study, we investigated amino acid sequence alignments between *Arabidopsis* ELP and their corresponding yeast ELP counterparts, revealing that AtELP5 and AtELP6 also share a conserved P-loop ATPase motif (Figure 3). In our yeast two-hybrid assays, we observed direct interactions between AtELP4 and AtELP6 and between AtELP5 and AtELP6 (Figure 1). Furthermore, our computational structure predictions in this study indicated that the structures of each *Arabidopsis* ELP4, ELP5, and ELP6 subunit exhibit a high degree of similarity to their corresponding yeast ELP subunits, forming a complex characterized by central, parallel, and twisted β-sheets flanked by α-helices (Figure 3). In more detail, both AtELP and their corresponding yeast Elp subunits exhibit a similar composition of central stranded β-sheets. For instance, AtELP4 contains 14 β-sheets, while ScElp4 has 14 (PDB ID 4A8J) [38] or 12 (PDB ID 4EJS) [47] β-sheets; AtELP5 has 10 strands, while ScElp5 has 9 (4A8J [38] and 4EJS [47]) β-sheets; and AtELP6 has 9 β-sheets, while ScElp6 has 9 (4A8J) [38] or 10 (4EJS) [47] β-sheets. Although both *Arabidopsis* and yeast subunits possess four additional β-sheets compared to the five-stranded β-sheets in RecA, a closer comparison of the structures of *Arabidopsis* and yeast ELP subunits revealed structural elements indicative of putative P-loop ATPase motifs in *Arabidopsis* ELP subunits that are highly similar to those found in RecA-like ATPases (Figure 3). When superimposing the *Arabidopsis* and yeast ELP456 complexes, our results indicate that the *Arabidopsis* ELP456 sub-complex shares a highly conserved tertiary structure with the yeast Elp456 sub-complex, despite the low sequence identity between each *Arabidopsis* ELP subunit and their corresponding yeast Elp counterparts (Figure 5).

Our complex modeling of AtELP456 further substantiated the in vivo experimental results, which demonstrated interactions between AtELP4 and AtELP6 and between AtELP5 and AtELP6 (Figure 1). It has been reported that the yeast Elp456 sub-complex consists of a homodimer of heterotrimers, forming a hexameric ring within the holo-Elongator complex [24,25]. Although we were unable to model the hexameric structure of the AtELP456 sub-complex, we predicted interactions between AtELP4 and AtELP5. These findings suggest the potential for the *Arabidopsis* ELP456 to also form a hexameric structure, similar to the yeast Elp456 sub-complex. Research groups including our own have previously noticed that despite overall low homology degrees among ELP123 and ELP456 sub-complexes from yeast to plants and animals, the ELP456 trimer does form a hetero-hexameric (ELP456)_2_ ring structure that associates with the catalytic ELP123 sub-complex. The specificity of this interaction suggests co-evolution between species, as demonstrated by pervious gene shuffle experiments [35,45]. The yeast Elp456 sub-complex is known to possess ATP-binding and ATPase catalytic sites, crucial for regulating tRNA binding by the Elongator complex [24,27]. In both yeast and *Arabidopsis*, the presence of similar defects in single mutants of each Elongator subunit suggests that all Elongator components contribute equally to the overall activity of the holo-Elongator complex [48,49].

Furthermore, we identified shared regions involved in establishing hydrogen bonds between *Arabidopsis* and yeast by comparing the positions of hydrogen bonds. This suggests that the structures of each ELP4, ELP5, and ELP6 protein, as well as the sub-complex, are conserved between *Arabidopsis* and yeast. These conserved motifs likely play crucial roles in the assembly of the ELP456 sub-complex and, potentially, the tRNA modification function of holo-Elongator.

Based on these computational analysis results, future investigations involving the mutagenesis of these residues within motifs hold the promise of shedding light on the biochemical functions and interactions related to ATP-mediated tRNA binding and the release of the AtELP456 sub-complex. Additionally, these findings are expected to offer fresh insights into the structure of the *Arabidopsis* holo-Elongator complex, thereby enhancing our understanding of the biochemical mechanisms governing the tRNA modifyingElongator complex and its targets in the regulation of mRNA translation during plant growth and development.

To further examine the evolutionary aspects of Elongator, rather than using wild-type loci of ELP4, ELP5, and ELP6 as in our study, such follow-up models could incorporate mutations previously reported as loss-of-functional alleles of ELP4, ELP5, and ELP6. These mutations have clinical relevance in cells from various organisms such as yeast, mice, and humans. Thus, this point provides valuable inspiration for a follow-up functional study on Elongator’s evolution and development.

## 4. Materials and Methods

### 4.1. Plant Materials and Growth Conditions

In this study, we utilized wild-type *Arabidopsis* Columbia-0 (Col-0) as our experimental model. Mutant lines including *Atelp4* (SALK_079193), *Atelp5* (SALK_143430), and *drl1-102* (SALK_056915) were sourced from the Arabidopsis Biological Resource Center (ABRC; https://abrc.osu.edu, Columbus, OH, USA, accessed on 18 July 2016). To ensure homozygous genotypes and generate double mutants, we followed the procedures outlined by Jun et al. [31]. For seed preparation, we carried out surface sterilization using sodium hypochlorite. Subsequently, seedlings were cultivated on Murashige–Skoog (MS) medium containing 2% sucrose and 0.2% gellan gum (pH 6.3) under long-day conditions (16 h of light/8 h of darkness at an intensity of 50–100 μE/m^2^/s) at 22 °C.

### 4.2. Yeast Two-Hybrid Assay and Yeast Growth Conditions

To investigate protein–protein interactions among Elongator subunits, we employed a GAL4-based ProQuest Two-Hybrid system (Invitrogen, Thermo Fisher Scientific, Waltham, MA, USA) for yeast two-hybrid assays. The cDNA corresponding to *Arabidopsis* Elongator subunits, specifically AtELP1 and AtELP4 through AtELP6, was amplified via PCR using gene-specific primers (Table 5) and subsequently cloned into the pENTR/D-TOPO vector. AtELP4 and AtELP6 were subcloned into the bait vector pDEST32 (DNA-binding domain (BD)), while AtELP1, AtELP3, AtELP5, and AtELP6 were subcloned into the prey vector pDEST22 (activation domain (AD)) using a Gateway LR clonase II system (Invitrogen, Thermo Fisher Scientific, Waltham, MA, USA). The yeast strain MaV203 (Invitrogen, Thermo Fisher Scientific, Waltham, MA, USA) underwent co-transformation with the aforementioned bait and prey constructs containing ELPs following the manufacturer’s guidelines. To assess interactions between the bait and prey, co-transformed yeast cells were streaked onto plates containing minimal SD medium devoid of leucine, tryptophan, and histidine. These plates were supplemented with either 0, 10, or 25 mM of 3-amino-1,2,4-triazole (3-AT) (Sigma-Aldrich, Taufkirchen, Germany). For the negative control, pDEST22 empty vectors were co-transformed with the pDEST32 constructs. Additionally, a β-galactosidase assay was conducted using co-transformed yeast cells, which were grown on SD/-Leu-Trp-His+3-AT medium supplemented with 500 μg/mL of 5-bromo-4-chloro-3-indolyl-β-D-galactopyranoside (X-gal, Sigma-Aldrich, Taufkirchen, Germany) at 30 °C.

### 4.3. Phylogeny Relationship and Motif Analysis

Eukaryote ELP5 full-length protein sequences were retrieved from the NCBI GenBank and Ensembl database and aligned using the ClustalX software with default parameters [50]. The phylogenic tree was constructed from aligned sequences of ELP5 using the neighbor-joining method (NJM). Conserved motifs of ELP5 were identified by using the MEME online tool (http://meme-suite.org/tools/meme, accessed on 14 December 2023) with the other default parameters and five maximum numbers of motifs [51]. The protein accession numbers of ELP5 are XP_004232350.1 (*Solanum lycopersicum*), XP_003615675.1 (*Medicago truncatula*), XP_003519251.3 (*Glycine max*), XP_031107344.1 (*Ipomoea triloba*), KAE8662382.1 (*Hibiscus syriacus*), XP_035544906.1 (*Juglans regia*), XP_021998395.1 (*Helianthus annuus*), KAG7036759.1 (*Cucurbita argyrosperma*), XP_021596387.1 (*Manihot esculenta*), XP_016560760.1 (*Capsicum annuum*), XP_033130608.1 (*Brassica rapa*), F4IQJ2.1 (*Arabidopsis thaliana*), XP_021862703.1 (*Spinacia oleracea*), XP_006357851.1 (*Solanum tuberosum*), XP_050113758.1 (*Malus sylvestris*), XP_006482241.1 (*Citrus sinensis*), XP_052481026.1 (*Gossypium raimondii*), XP_023770943.1 (*Lactuca sativa*), XP_022894256.1 (*Olea europaea* var. sylvestris), XP_021827752.1 (*Prunus avium*), XP_019262098.1 (*Nicotiana attenuate*), XP_011462619.1 (*Fragaria vesca* subsp. vesca), XP_021643885.2 (*Hevea brasiliensis*), XP_010229016.1 (*Brachypodium distachyon*), XP_025793170.1 (*Panicum hallii*), XP_015632160.1 (*Oryza sativa* Japonica Group), NP_001144011.2 (*Zea mays*), XP_044982550.1 (*Hordeum vulgare* subsp. Vulgare), XP_048572141.1 (*Triticum Urartu*), XP_002468319.1 (*Sorghum bicolor*), XP_031476161.1 (*Nymphaea colorata*), XP_042502504.1 (*Macadamia integrifolia*), XP_057818170.1 (*Cryptomeria japonica*), XP_024526123.1 (*Selaginella moellendorffii*), XP_024375976.1 (*Physcomitrium patens*), A1A5V9.1 (*Danio rerio*), Q99L85.1 (*Mus musculus*), Q8TE02.2 (*Homo sapiens*), XP_003131977.1 (*Sus scrofa*), XP_005597795.1 (*Equus caballus*), XP_027694435.1 (*Vombatus ursinus*), XP_039766820.1 (*Ornithorhynchus anatinus*), XP_003229953.3 (*Anolis carolinensis*), XP_042703699.1 (*Chrysemys picta bellii*), XP_058054886.1 (*Anopheles bellator*), XP_018095279.1 (*Xenopus laevis*), Q24050 (*Drosophila melanogaster*), XP_060816056.1 (*Bombus pascuorum*), XP_029156538.1 (*Nylanderia fulva*), XP_001814270.1 (*Tribolium castaneum*), NP_001309581.1 (*Caenorhabditis elegans*), XP_020914534.1 (*Exaiptasia diaphana*), XP_012553489.1 (*Hydra vulgaris*), CAI8038430.1 (*Geodia barrette*), KAJ8021666.1 (*Holothuria leucospilota*), P38874.1 (*Saccharomyces cerevisiae*), QGN13721.1 (*Kluyveromyces marxianus*), and O94495.1 (*Schizosaccharomyces pombe*).

### 4.4. Structure Prediction

We obtained the structural predictions of *Arabidopsis* ELP subunits, generated by AlphaFold2 (https://colab.research.google.com/github/sokrypton/ColabFold/blob/main/AlphaFold2.ipynb, accessed on 18 October 2022), from the AlphaFold Protein Structure Database [52] (available at https://alphafold.ebi.ac.uk/, accessed on 18 October 2022) with the following accession codes: AF-Q9C778 for AtELP4, AF-F4IQJ2 for AtELP5, and AF-Q8L9Y2 for AtELP6. Structural alignment was conducted using the RCSB Pairwise Structure Alignment tool (https://www.rcsb.org/alignment, Piscataway, NJ, USA, accessed on 24 October 2022). For the prediction of complex structures involving *Arabidopsis* ELP subunits, we utilized AlphaFold2 at the ColabFold Google Colab notebook [53] (available at https://colab.research.google.com/github/sokrypton/ColabFold/blob/main/AlphaFold2.ipynb, accessed on 27 October 2022; refer to Figure 3). To visualize the predicted structures, we employed iCn3D (https://www.ncbi.nlm.nih.gov/Structure/icn3d/, accessed on 3 November 2022) [54] and the Mol*3D viewer in RCSB PDB (https://www.rcsb.org/3d-view, accessed on 24 October 2022) [55].

## Figures and Tables

**Table 1:** Amino acid sequence homology between *Arabidopsis* and yeast.

Yeast	*Arabidopsis*	AGI	Homology (%)	Function
ELP1/ELO2/TOT1/KTI7/IKI3	AtELP1	AT5G13680	27	Apoenzyme
ELP2/TOT2/KTI3	AtELP2	AT1G49540	29	Apoenzyme
ELP3/ELO3/TOT3/KTI8	AtELP3	AT5G50320	63	Coenzyme; histone acetyl transferase
ELP4/ELO1/TOT7/KTI9	AtELP4	AT3G11220	20	Apoenzyme
TLP5/TOT5/IKI1	AtELP5	AT2G18410	12	Apoenzyme
ELP6/TOT6/KEI4	AtELP6	AT4G10090	16	Apoenzyme
ELO4/KTI12/TOT4	DRL1	AT1G13870	28	

**Table 2:** Structural similarity of *Arabidopsis* and yeast Elp4, 5, and 6 proteins assessed using the RCSB pairwise structure alignment tool.

Protein	Reference (*Arabidopsis*)	Target (Yeast)	RMSD	TM-Score	Sequence Identity	Equivalent Residues	Reference Coverage	Target Coverage
ELP4	AF-Q9C778	4A8J_A	3.08	0.65	20%	261	74%	94%
ELP5	AF-F4IQJ2	4A8J_B	3.05	0.43	11%	187	50%	85%
ELP6	AF-Q8L9Y2	4A8J_C	3.05	0.74	14%	230	88%	87%

**Table 3:** Key residues involved in hydrogen bonds at the ELP4–6 and ELP5–6 interfaces in *Arabidopsis* and yeast. Hydrogen bonds in *Arabidopsis* ELPs were predicted using iCn3D, with default values for the distance between the hydrogen atom and the acceptor atom set below 0.38 nm. Hydrogen bonds in yeast ELP456 sub-complex were referenced from [25].

	In the Interface between AtELP4–AtELP6			In the Interface between ScElp4–ScElp6		In the Interface between AtELP5–AtELP6			In the Interface between ScElp5–ScElp6	
	AtELP4	AtELP6	Distance (nm)	ScElp4	ScElp6	AtELP5	AtELP6	Distance (nm)	ScElp5	ScElp6
1	ASN(N)32:ND2	GLU(E)250:OE1	0.37	GLU(E)303:OE1	SER(S)159:HG	TRP(W)100:O	ARG(R)119:NH2	0.32	TYR(Y)36:OH	PRO(P)244:N
2	GLN(Q)180:OE1	LYS(K)100:NZ	0.36	LYS(K)320:NZ	ASP(D)111:OD1	TRP(W)100:N	TYR(Y)164:OH	0.32	SER(S)59:OG	ARG(R)176:NH2
3	SER(S)231:OG	LEU(L)97:O	0.26	LYS(K)320:NZ	ASP(D)111:OD2	TYR(Y)94:OH	HIS(H)166:ND1	0.31	ASP(D)74:OD1	ARG(R)176:NH1
4	ARG(R)256:NH1	GLU(E)147:OE2	0.33	ASN(N)346:OD1	ASN(N)186:ND2	TYR(Y)94:OH	SER(S)170:OG	0.36	ASP(D)74:OD2	ARG(R)176:NE
5	ARG(R)256:NH2	GLU(E)147:OE2	0.20	ASN(N)346:OD1	GLN(Q)150:NH2	ASP(D)231:OD1	SER(S)237:OG	0.27	ASP(D)74:OD2	ARG(R)176:NH1
6	HIS(H)259:ND1	HIS(H)181:ND1	0.36	ASN(N)346:OD1	GLN(Q)150:O	ASP(D)231:OD2	SER(S)237:OG	0.37	TYR(Y)111:OH	ASN(N)167:ND2
7	HIS(H)259:NE2	ASP(D)183:OD	0.26	ASN(N)346:ND2	GLN(Q)150:NE2	PHE(F)232:N	ASN(N)239:O	0.34	TYR(Y)111:OH	SER(S)210:OG
8				ASN(N)346:ND2	GLN(Q)150:OE1	GLN(Q)230:O	LEU(L)241:N	0.26	LYS(K)140:O	ASN(N)206:ND2
9						GLN(Q)230:N	LEU(L)241:O	0.28	LYS(K)140:N	ASN(N)206:ND2
10						VAL(V)228:O	ASN(N)243:N	0.30	LYS(K)140:N	ASN(N)206:O
11						HIS(H)229:NE2	TYR(Y)257:OH	0.29	LYS(K)140:NZ	GLN(Q)205:OE1
12									ARG(R)195:NH1	GLU(E)258:OE1
13									ARG(R)195:NH2	GLU(E)258:OE2
14									ASN(N)198:OD1	ARG(R)240:NH1
15									ASN(N)198:ND2	ARG(R)240:NH1
16									ASN(N)199:OD1	ARG(R)240:NH2
17									ASN(N)199:OD1	ARG(R)240:NH1

**Table 4:** Key residues involved in hydrogen bonds at the interface of the homodimeric complexes of AtELP456 and yELP456 sub-complexes. Hydrogen bonds were predicted using iCn3D, with default values for the distance between the hydrogen atom and the acceptor atom set below 0.38 nm. Hydrogen bonds in the yELP456 sub-complex were referenced from [25].

	In the Interface between AtELP4–AtELP5			In the Interface between ScElp4–ScElp5	
	AtELP4	AtELP5	Distance(nm)	ScElp4	ScElp5
1	PRO(P)211:O	PRO(P)163:N	0.38	GLU(E)117:N	SER(S)212:O
2	PRO(P)211:O	SER(S)166:OG	0.38	THR(T)116:O	GLY(G)213:O
3	SER(S)247:O	ASN(N)196:ND2	0.30	THR(T)116:O	GLY(G)213:N
4	HIS(H)68:NE2	GLY(G)246:O	0.29	THR(T)116:N	ARG(R)214:N
5	TYR(Y)284:O	ARG(R)247:NH1	0.36	THR(T)337:OG1	ASN(N)153:O
6	ILE(I)270:O	VAL(V)248:N	0.31	SER(S)304:OG	ASN(N)153:ND2
7	ASP(D)272:N	VAL(V)248:O	0.26	SER(S)304:N	ASN(N)153:ND2
8	LYS(K)268:NZ	SER(S)279:O	0.33	PHE(F)302:O	ASN(N)153:ND2

**Table 5:** Sequences of oligonucleotides used in this study.

Name	Sequence	Tm Value (°C)	Purpose
ELP1-forward	CACCATGAAAAGAGATGAAGATTTGAC	51.3	cloning
ELP1-reverse	TCATGGGCTTATGAAGACCT	53.4	cloning
ELP2-forward	CACCATGTCAGAAAACACAAAAGTCGA	53.1	cloning
ELP2-reverse	TCAAAACTTGAAGTTAAAAACTCTC	52.4	cloning
ELP3-forward	CACCATGGCGACGGCGGTAG	54.0	cloning
ELP3-reverse	TCAAAGAAGATGCTTCACCA	51.3	cloning
ELP4-forward	CACCATGGCTGCACCAAACGTTC	54.9	cloning
ELP4-reverse	TCAAAAATCTAGTGCTCCGG	53.4	cloning
ELP5-forward	CACCATGGCGGAATCGATTTTCAG	53.4	cloning
ELP5-reverse	TTAAATGTCCAAATCATCATCAGGA	54.0	cloning
ELP6-forward	CACCATGGATCGTTCTTTGAATCTC	51.9	cloning
ELP6-reverse	TCAGCTTCTGCAACCAGGAT	55.4	cloning
DRL1-forward	CACCATGGCGCTAGTTGTGATTTG	53.4	cloning
DRL1-reverse	TCAAGCGTTATTACCTCCAAAC	54.4	cloning

## Data Availability

The datasets presented in this study can be found in online repositories. The names of the repository/repositories and accession number(s) can be found in the article.
